# NRN1 interacts with Notch to increase oncogenic STAT3 signaling in melanoma

**DOI:** 10.1186/s12964-024-01632-8

**Published:** 2024-05-06

**Authors:** Lucia Devitt, Dana Westphal, Katharina Pieger, Nadja Schneider, Anja Katrin Bosserhoff, Silke Kuphal

**Affiliations:** 1 Institute of Biochemistry, Friedrich-Alexander-University Erlangen-Nürnberg, Fahrstrasse 17, Erlangen, 91054 Germany; 2 Department of Dermatology, Faculty of Medicine and University Hospital Carl Gustav Carus at TU Dresden, Dresden, Germany; 3 National Center for Tumor Diseases (NCT) Dresden, a partnership between German Cancer Research Center (DKFZ), Faculty of Medicine and University Hospital Carl Gustav Carus at TU Dresden, and Helmholtz-Zentrum Dresden - Rossendorf (HZDR), Dresden, Germany

**Keywords:** NRN1, Notch, STAT3, Melanoma

## Abstract

**Background:**

Melanoma is a highly heterogeneous cancer, in which frequent changes in activation of signaling pathways lead to a high adaptability to ever changing tumor microenvironments. The elucidation of cancer specific signaling pathways is of great importance, as demonstrated by the inhibitor of the common BrafV600E mutation PLX4032 in melanoma treatment. We therefore investigated signaling pathways that were influenced by neurotrophin NRN1, which has been shown to be upregulated in melanoma.

**Methods:**

Using a cell culture model system with an NRN1 overexpression, we investigated the influence of NRN1 on melanoma cells’ functionality and signaling. We employed real time cell analysis and spheroid formation assays, while for investigation of molecular mechanisms we used a kinase phosphorylation kit as well as promotor activity analysis followed by mRNA and protein analysis.

**Results:**

We revealed that NRN1 interacts directly with the cleaved intracellular domain (NICD) of Notch1 and Notch3, causing a potential retention of NICD in the cytoplasm and thereby reducing the expression of its direct downstream target Hes1. This leads to decreased sequestration of JAK and STAT3 in a Hes1-driven phosphorylation complex. Consequently, our data shows less phosphorylation of STAT3 while presenting an accumulation of total protein levels of STAT3 in association with NRN1 overexpression. The potential of the STAT3 signaling pathway to act in both a tumor suppressive and oncogenic manner led us to investigate specific downstream targets – namely Vegf A, Mdr1, cMet - which were found to be upregulated under oncogenic levels of NRN1.

**Conclusions:**

In summary, we were able to show that NRN1 links oncogenic signaling events between Notch and STAT3 in melanoma. We also suggest that in future research more attention should be payed to cellular regulation of signaling molecules outside of the classically known phosphorylation events.

**Graphical Abstract:**

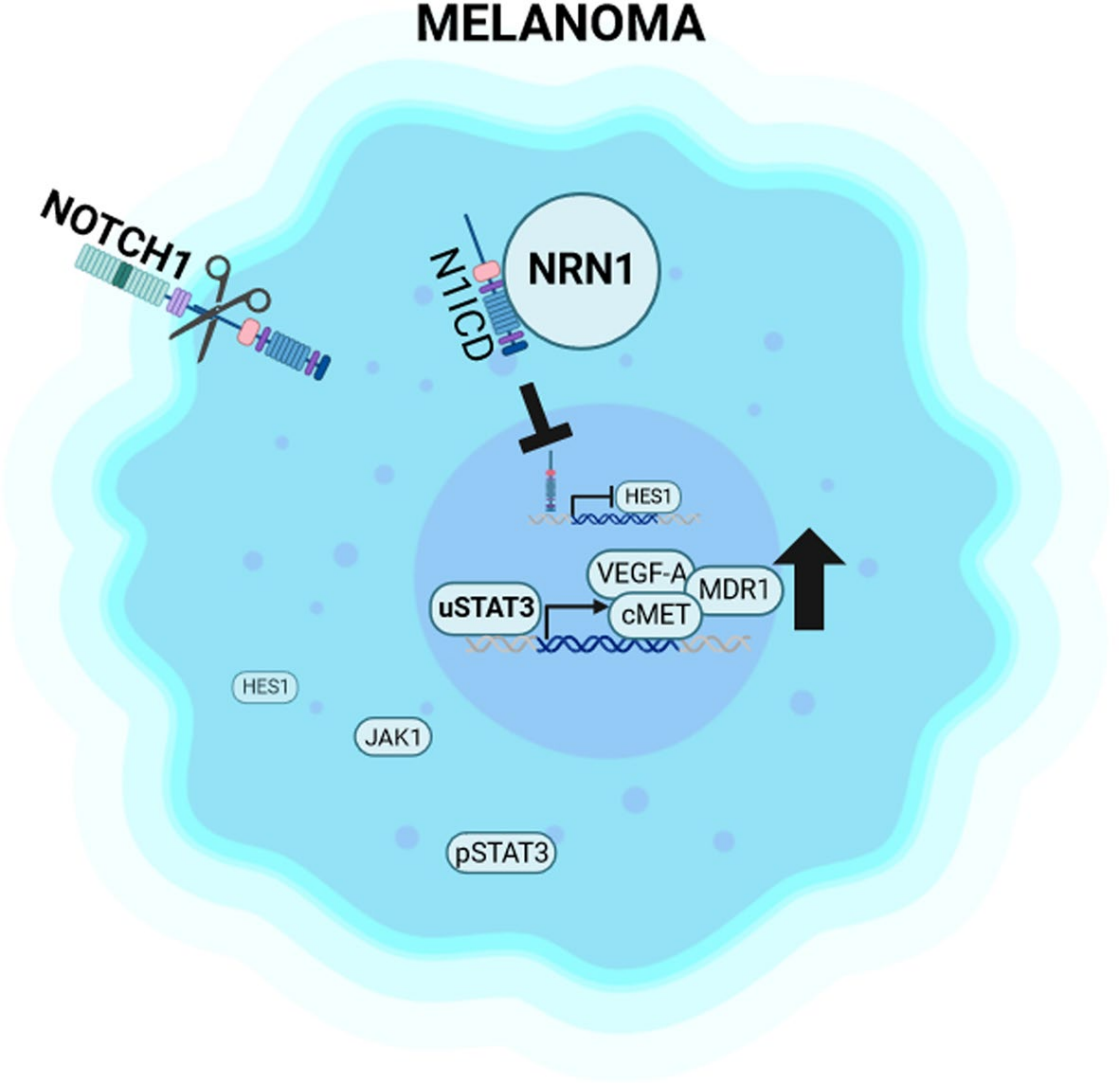

**Supplementary Information:**

The online version contains supplementary material available at 10.1186/s12964-024-01632-8.

## Introduction

Malignant melanoma is known to be one of the most aggressive forms of skin cancer, mostly due to its very early metastasis, which often occurs in the brain [1]. The most important factor in survivability still remains early detection. A major breakthrough in melanoma treatment was the suite of kinase inhibitors, most notably the specific BrafV600E inhibitor PLX4032 (trade name Vemurafenib) and the immune checkpoint inhibitors which target PD-1 or CTLA-4 [2]. Due to developing resistance mechanisms during kinase inhibitor treatment or primary resistance in the case of immune checkpoint inhibitors, the search for new potential targets and an overall better understanding of melanoma development and progression is ongoing.

One approach is to investigate molecules that are important for the shared embryological cell fate between melanoblasts (the later melanocytes and originators of melanoma) and neurons, since both cell types arise from multipotent neural crest cells [3]. Furthermore, the network of involved signaling molecules is very similar between events of embryogenesis and the development and progression of malignant melanoma. One promising molecule is Neuritin-1 (NRN1, also named candidate plasticity gene 15), a member of the neurotrophin family known primarily from the brain. The cpg15 gene encodes the highly conserved protein NRN1 which is 142-amino acids (aa) long. It contains both a predicted 27-aa secretory signal peptide at its N-terminus and a glycosylphosphatidylinositol anchor at its C-terminus (aa 116). NRN1’s function was originally established for neuroprotection and regeneration [4, 5]. However, other described functions of NRN1 are also related to the research field of oncology. There is recent evidence that NRN1 expression may be upregulated in different tumor entities [6–9]. In a previous publication, we already investigated the role of NRN1 in melanoma functionally, both *in vitro* and *in vivo*. For melanoma we showed that NRN1 is involved in regulating migration, attachment-independent growth, and vascular mimicry [10]. Although some data has been assembled on NRN1 in physiological and pathophysiological processes and for nervous system development and plasticity, few details exist on the possible presence of a membrane-bound receptor or intracellular signal transduction [11]. NRN1 may be a potential ligand for the insulin receptor (IR) or the insulin-like growth factor type 1 receptor (IGF-1R) in granule neurons [12]. Furthermore, a co-localization of NRN1 to the tyrosine kinase A (TrkA) receptor was shown in dorsal root ganglia neurons [13]. To date, it is completely unclear whether NRN1 requires a receptor at all for signal transduction or whether its functions may be controlled purely from the cytoplasm. No receptor for NRN1 has yet been discovered in melanoma either.

Recently Zhang et al. defined a role for NRN1 in the Notch signaling pathway. NRN1 interacts with the molecule Neuralized E3 ubiquitin protein ligase 1 which inhibits Notch signaling and promotes neurite growth [14]. Notch signaling is ubiquitously found in all animal species and plays a major role in early development and cell fate determination. In humans – and all other mammals – the signaling is achieved through 4 different receptors (Notch1-4) and 5 ligands (Delta-like 1, 3, 4; Jagged 1, 2), which are all located in the plasma membrane as transmembrane proteins. The interactions between receptor and ligand occur extracellularly, while the signal transduction occurs through cleavage of the Notch intracellular domain (NICD) and its translocation into the nucleus for transcriptional activation of defined genes [15, 16]. In the nucleus NICD interacts with a transcriptional activation complex mainly comprised of the DNA-binding molecule RBP-J (also termed CBF1) and Mastermind-like (MAML) protein. In this case e.g. hairy and enhancers of split (HES) family genes have been identified as downstream effector genes. Although RBP-J is accepted as the major effector for the canonical Notch, RBP-J independent non-canonical Notch signaling has also reported [17]. Notch signaling is known to contribute to melanoma progression [18], acting both as a tumor promoter or suppressor depending on the cellular context [19–22]. Mikheil et al. showed that forced expression of NICD, the active form of Notch, was sufficient to induce apoptosis independently of MAPK pathway inhibition in melanoma cells, with both intrinsic and acquired resistance to MAPK inhibitors [21]. Considering the oncogenic role of Notch1 signaling in melanoma, a phase II trial using broad Notch signaling inhibitors surprisingly only showed minimal clinical activity against metastatic melanoma [19]. In general, melanoma research to date has been clearly focused on the analysis of Notch1 [23].

Current literature from oncology and neurology research links the Notch/HES1 pathway with family of signal transducers and activators of transcription (STAT)-dependent signaling pathways. In particular, a direct protein interaction between HES1 and JAK/STAT has been postulated [23], and in glioblastoma multiforme it has been shown that inhibition of STAT leads to activation of Notch [24]. In the past, STAT signaling has been considered as activated upon phosphorylation of the STATs (pSTATs) at distinct sites through extracellular molecules and ligands like growth factors and cytokines [25]. Activated (p)STAT1 and (p)STAT3 have often been assigned a role as oncogenic factors, including in melanoma [26, 27] whereas in prostate, lung and colorectal carcinomas, tumor suppressive roles were associated with STAT3 function depending on the mutational context [28–31]. Interestingly, evidence has emerged of a potential second wave, phosphorylation-independent (non-canonical) signaling pathway induced by STATs [32]. Here, unphosphorylated STATs (uSTATs, no Y705 or S727 phosphorylation) can shuttle in and out of the nucleus without phosphorylation playing a role [33]. In the nucleus uSTATs bind to NFκB subunit p65 (also unphosphorylated) and corresponding NFκB-DNA consensus sequences or to the DNA motif M67 (a modified c-fos cis-inducible enhancer sequence) or to the gamma interferon activation site (GAS) promoter sequences (generically TT_4-5N_AA) and are part of transcriptional processes [34–36]. Some of the targets are unique to uSTAT and some of them overlap with pSTAT signaling [35, 37]. In addition, the literature already provides evidence for specific target genes of uSTAT, especially uSTAT3. Mras, Met, Rantes, IL6, and IL8 are targets of uSTAT3 after stimulation of the cells with IL6 [35], and for breast cancer and head and neck squamous cell carcinoma cyclinB1 and E2f1 were also described as targets of uSTAT3 [38]. In other investigations Jun activation domain-binding protein 1 (JAB1) was identified as a direct binding partner of uSTAT3, but not of pSTAT3, in the nucleus of colon carcinoma cells. Here the target genes were defined as Mdr1, Nanog, and Vegf [39].

Our latest research results link the functions of intracellular NRN1 to the Notch and STAT3 signaling pathways in melanoma. We detected a retention of Notch-NICD in the cytoplasm of melanoma cells through interaction with NRN1. NRN1 then leads to extenuated translocation of NICD into the nucleus with the consequence of reduced expression of Hes1. The absence of Hes1 potentially results in decreased sequestration of JAK and STAT3 in a Hes1-driven phosphorylation complex. Consequently, our data show less phosphorylation of STAT3 but rather accumulation of the total protein of STAT3 in melanoma. In summary, NRN1 is involved in the maintenance of oncogenic STAT3 signaling.

## Materials and Methods

### Cell lines and cell culture conditions

All cell lines (Table 1) were incubated at 37 °C in an 8 % CO_2_ humidified atmosphere [40]. All human cell lines have been authenticated using Short Tandem Repeat (STR) profiling within the last three years (DSMZ, Braunschweig, Germany and Multiplexion, Heidelberg, Germany). All experiments were performed with mycoplasma-free cells (MycoSEQ mycoplasma detection system, Thermo Fisher Scientific, Waltham, Massachusetts, USA). Mel JuSo (RRID:CVCL_1403) and Mel JuSo cell clones (GFP, NRN1-GFP) were cultivated in RPMI 1640 with 2 % sodium bicarbonate.

**Table 1:** Cell lines used for experiments including official identifiers and sources. Additional information about growth media and supplements for each cell line

**Cell Line**	**Resource Identification Initiative** **(Cellosaurus)**	**Source**
MCF-7	CVCL_0031	ATCC, HTB-22
SK-MEL-28	CVCL_0526	ATCC, HTB-72
Mel JuSo	CVCL_1403	DSMZ, ACC 74
HEK293T	CVCL_0063	?
SBcl2	CVCL_D732	Meenhard Herlyn The Wistar Institute, Philadelphia, USA
WM9	CVCL_6806	

*ATCC* American Type Culture Collection, *DSMZ* Leibniz Institute DSMZ-German Collection of Microorganisms and Cell Cultures

MCF-7, SK-MEL-28, HEK293Z were cultivated in Dulbecco’s modified Eagle’s medium (DMEM) supplemented with penicillin (400 units/ml), streptomycin (50 μg/ml) and 10 % fetal calf serum (all from Sigma-Aldrich, Steinheim, Germany). Mel JuSo cell lines were cultivated in Roswell Park Memorial Institute 1640 (RPMI 1640) medium supplemented with 2 % sodium bicarbonate, penicillin (400 units/ml), streptomycin (50 μg/ml) and 10 % fetal calf serum (all from Sigma-Aldrich, Steinheim, Germany)

https://wistar.org/research-discoveries/business-development/research-tools

Wistar cell lines were cultivated in MCDB153 (Sigma-Aldrich) with 20 % Leibovitz’s L-15 (PAA Laboratories, Coelbe, Germany), 2 % FCS, 1.68 mM CaCl_2_ (Sigma-Aldrich), and 5 µg/ml insulin (Sigma-Aldrich)

### Paired organ/brain melanoma metastasis samples

Samples of paired organ and brain melanoma metastases were provided by Dana Westphal. The pair ZueMel1H/ZueMel1 was a gift from Reinhardt Dummer to Heike Niessner, while TueMel32/TueMel32H were established by Heike Niessner [41]. The third pair was established as mentioned before [42].

### Proteome Profiler Human Phospho-Kinase Array Kit

The Phospho Proteome Profiler Kit (CAT# ARY003B; R&D Systems Inc, Minneapolis, USA) was performed as described in the manufacturer’s instructions using 200 μg protein lysate.

### RNA isolation and reverse transcription

Isolation of total cellular RNA from cultured cells was performed with the E.Z.N.A.® Total RNA Kit I (Omega Bio-Tek, Norcross, GA, USA) according to the manufacturer’s instructions.

Generation of cDNAs by reverse transcription (RT) reaction was performed with the Superscript® II Reverse Transcriptase Kit (Thermo Fisher Scientific Inc, MA, USA) which was used according to the manufacturer’s instructions.

### Analysis of mRNA expression

For the quantitative real time polymerase chain reaction (qRT-PCR) the Lightcycler® II 480 SYBR Green I Master Kit (Roche Diagnostics GmbH, Mannheim, Germany) was used. The PCR products formed were detected with SYBR Green I dye. The qRT-PCR was performed in 96-well microtiter plates (LightCycler® 480 Multiwell Plate 96, Roche Diagnostics GmbH) for each gene of interest in duplicates. qRT-PCR analysis of gene expression was performed on a LightCycler 480 system (Roche Diagnostics GmbH). Specific sets of primer sequences are listed in Table 2.

**Table 2:** List of primer pairs used for qRT-PCR. Primer names with position number of RefSeq sequence, along with amino acids of forward (for) and reverse (rev) sequences

**Primer Name**	**Sequence for (5‘-3‘)**	**Sequence rev (5‘-3‘)**
hActinb 735	CTACGTCGCCCTGGACTTCGAGC	
hActinb 1119		TGGAGCCGCCGATCCACACGG
hNrn-1 for786	GGGCGACAGCATGGCCAACT	
hNrn-1 rev992		CCGCTGCCGCAGAGTTCGAA
hHes-1for_116	CCTCAGCACTTGCTCAGTAGTT	
hHes-1rev_411		TCAGCTGGCTCAGACTTTCAT
hHey1 for1710	AGTTAGGAGAGAGCCGCTGA	
hHey1 rev1896		AATTGACCACTCGCACACCA
Vegf A for	CAGCGCAGCTACTGCCATCCAATCGAGA	
Vegf A rev		GCTTGTCACATCTGCAAGTACGTTCGTTTA
hMdr1 for3534	CCAGAAACAACGCATTGCCA	
hMdr1 rev3821		GCCTGGACACTGACCATTGA
hcMet for4152	AACCCGAATACTGCCCAGAC	
hcMet rev4342		AGAAGGATACGGAGCGACAC

### siRNA and plasmid transfection

All transient transfections were performed in 6-well plates. For knockdowns using siNRN1 pool (Gene ID: 51299, siTools Biotech GmbH, Planegg, Germany) 1.5 x 10^5^ cells were transfected with 5 nM siNRN1 pool while floating according to the Lipofectamine^TM^ RNAiMAX (Thermofisher Scientific) protocol. Cells were then incubated for 48 h and harvested. Plasmid (Table 3) transfections were performed on sitting cells (4 h or 16 h after seeding) using the Lipofectamine^TM^ LTX Plus system (Invitrogen, Darmstadt, Germany) according to the provided protocol. Plasmid transfected cells were incubated for 24 - 48 h depending on the construct and subsequently harvested or used in further experiments.

**Table 3:** List of plasmids used for transduction or transfection of cells. Company of origin, producer and reference publications included along with description of vector content

**Vector**	**Company**	**Producer**	**Ref.**	**Description**
pCMV-NRN1-GFP	Backbone and insert from Origene, Rockville, USA	Lucia Devitt	-	Lentiviral NRN1 overexpression construct, under CMV promotor, with GFP tag
pHes1(467)-luc	RRID:Addgene_41723	Ryoichiro Kageyama & Raphael Kopan	[43]	~513 bp of the Hes1 promoter in front of the luciferase gene
pMDR1-1202	RRID:Addgene_37627	Kathleen Scotto	[44]	MDR1 promoter in front of the luciferase gene
pGL4.10-VEGFprom	RRID:Addgene_66128	David Mu	[45]	-1 000 to -1 bp of the Vegf A promoter in front of the luciferase gene
Notch1 intracellular domain-pcw107-V5	RRID:Addgene_64622	David Sabatini & Kris Wood	[46]	Physiological expression of N1ICD tagged with V5
pRL-TK	Promega, Madison, USA	-	-	Renilla luciferase under HSV-thymidine kinase promotor

### Transfection experiments and reporter gene assay

1.5 x 10^5^ cells/well were seeded into 6-well plates and transfected with 0.5 µg plasmid DNA using the Lipofectamine^TM^ LTX Plus method according to the manufacturer’s instructions. The luciferase plasmids were used for the reporter gene assay. Transfection efficiency was normalized to renilla luciferase activity by cotransfecting 0.1 µg of the plasmid pRL-TK (Promega, Mannheim, Germany). The cells were lysed 16 h after transfection and luciferase activities were determined. pHes1(467)-luc was a gift from Ryoichiro Kageyama & Raphael Kopan (Addgene plasmid # 41723; http://n2t.net/addgene:41723 ; RRID:Addgene_41723) [43]. pMDR1-1202 was a gift from Kathleen Scotto (Addgene plasmid # 37627; http://n2t.net/addgene:37627 ; RRID:Addgene_37627) [47]. pGL4.10-VEGFprom(-1000 to -1) was a gift from David Mu (Addgene plasmid # 66128; http://n2t.net/addgene:66128 ; RRID:Addgene_66128) [45]. Notch1 intracellular domain-pcw107-V5 was a gift from David Sabatini & Kris Wood (Addgene plasmid # 64622; http://n2t.net/addgene:64622 ; RRID:Addgene_64622) [46].

### Western blot

As described previously [26], cell pellets were lysed in 100 µl Radioimmunoprecipitation Assay buffer (RIPA) for 15 min at 4 °C. Cell fragments were removed by centrifugation (13 000 rpm, 10 min, 4 °C), and the supernatants were collected. 20 to 40 µg of total RIPA lysates were loaded onto polyacrylamide gels. After separation, the gel was blotted onto a PVDF membrane, and blocked for 1 h with either 5 % milk powder (MP)/TBS-T or 5 % bovine serum albumin (BSA)/TBS-T. Membranes were incubated in primary antibodies (Table 4) overnight at 4 °C, diluted in 5 % MP/TBS-T or 5 % BSA/TBS-T. After washing three times with TBS-T, the membrane was incubated with a horseradish peroxidase (HRP)-coupled secondary antibody (anti-rabbit HRP or anti-mouse HRP, Cell Signaling Technology; 1 in 2 000 dilution in TBS-T) for 1 h. The immunoreactions were visualized by ECL staining (Bio-Rad, Feldkirchen, Germany). The densitometry was measured using the LabImage software (Kapelan Bio-Imaging, Leipzig, Germany).

**Table 4:** List of antibodies used for Western blot and immunofluorescence experiments. Manufacturing company listed along with specific reference, as well as working solution dilutions

**Antibody**	**Company**	**Reference**	**Dilution (WB unless otherwise stated)**
ACTINB	Sigma Aldrich, Steinheim, Germany	A5541	1 in 5 000
Alexa 555 Plus	Invitrogen, Paisley, UK	A32727	1 in 500
Alexa 647 Plus	Invitrogen	A32733/A32728	1 in 500
GAPDH	Cell Signaling Technology, Frankfurt a. M. Germany	#2118	1 in 1 000
GFP	ThermoFisher Scientific, Dreieich, Germany	A-11122	1 in 2 000
HES1	Cell Signaling Technology	#11988	1 in 1 000
LAMINB2	Merck KGaA, Darmstadt, Germany	MAB3536	1 in 1 000
NEURITIN 1 (NRN1)	Santa Cruz Biotechnology, Heidelberg, Germany	sc-365538	WB: 1 in 1 000 IF: 1 in 100
Notch3	Cell Signaling Technology	#5276	1 in 1 000
N1ICD	Cell Signaling Technology	#4147	1 in 1 000
STAT1	Santa Cruz Biotechnology	sc-464	WB: 1 in 1 000 IF: 1 in 200
Phospho-STAT1	Santa Cruz Biotechnology	sc-8394	WB: 1 in 1 000 IF: 1 in 100
STAT3	Cell Signaling Technology	#9139	WB: 1 in 1 000 IF: 1 in 500
Phospho-STAT3	Cell Signaling Technology	#9145	WB: 1 in 1 000 IF: 1 in 500
V5	Cell Signaling Technology	#13202	1 in 1000

### Immunoprecipitation (IP)

For immunoprecipitation experiments, cells were transfected with relevant plasmids (pcDNA, N1ICD-V5) and harvested after 48 h. Proteins were harvested using standard RIPA buffer lysis and prepared as 1 μg/μl solutions in PBS. To avoid unspecific binding events, protein lysates were precleared with Protein G-Sepharose beads (VWR International, 17-0618-01) for 3 h at 4 °C while rotating. Afterwards the beads were discarded and supernatants harvested by centrifugation (1 min at 2 000 rpm, 4 °C). Supernatants were incubated with 0.8 μg antibodies at 4 °C overnight while rotating, then 25 μl beads were added for another overnight incubation as before. Beads bound with antibody and proteins of interest were harvested by centrifugation as before, while supernatants were discarded. Antibody-protein complexes were dissociated from beads using 30 μl reducing Lämmli buffer at 95 °C for 5 min. Proteins were analysed using polyacrylamide gels as described above. As secondary antibody, Veriblot (ab131366, abcam) was used at 1 in 500 dilution in 5 % MP/TBS-T.

### Enzyme linked immunosorbent assay

The ELISA was performed as described in the manufacturer’s instructions (CAT #CSB-EL016088HU; Hölzel Diagnostika GmbH, Cologne, Germany).

### Overexpression construct and lentiviral transduction

Packaging cells (HEK293T) were transfected with a 3‐plasmid system. For transfections, 12 μg pCMVΔR8.2, 6 μg pHIT G, and 12 μg plasmid DNA of interest (pCMV-GFP and pCMV-NRN1-GFP) were combined with DMEM (without phenol red) and subsequently mixed with 24 μl Lipofectamine Plus (Invitrogen) to a final volume of 160 μl (mixture A). Twenty microliters of Lipofectamine LTX (Invitrogen) were mixed with 140 μl DMEM (without phenol red) and incubated for 10 min (mixture B). After incubation, mixtures A and B were combined, incubated for 30 min at RT, and finally added to HEK293T cells, which were seeded the previous day in 10 ml high glucose DMEM into a 10 cm dish. Twenty‐four hours later, lentiviral supernatants were collected and filtered (0.45 μm pore size) for the subsequent infection of target cells (Mel JuSo). Harvested pCMV-NRN1-GFP supernatants were mixed with 1 volume Lenti Concentrator (Origene, cat# TR30025) for every 4 volumes supernatant. Following incubation at 4 °C for 6 h, viral particles were harvested through centrifugation at 3 500 g, 4 °C for 25 min and resuspended in 6 ml. The concentrated viral solutions were then added to the cells of interest for infection. The infected cells were incubated for 16 h, and the medium was subsequently changed to remove remaining virus particles.

### Nuclear and cytoplasmic separation of protein

For subcellular fractionation, 1 x 10^6^ cells were seeded in a T125 culture flask and detached by scraping after two days. In the case of RNAi-knockdown, floating cells were transfected with Lipofectamine™ RNAiMAX (Thermofisher Scientific, Grand Island, New York, NK, USA) and medium was changed the next day. To separate nuclear and cytosolic proteins, cells were homogenized and fractionated as previously described [48].

### Proliferation with the xCELLigence system

The xCELLigence System (Roche Diagnostics GmbH, Mannheim, Germany) is based on measurement of electrical impedance and permits real-time analysis of migration, and proliferation. E-plates were used and basic protocols recommended by the manufacturer were followed. 4 x 10^2^ cells/well were counted for the proliferation measurement. Impedance is represented by the relative and dimensionless parameter named cell index (CI). CI values = Z_i_-Z_0_/15[Ohm]; where Z_0_ = impedance at the start of the experiment, and Z_i_ = impedance at individual time points during the experiment. The normalized cell index (NCI) was calculated as the cell index CI_ti_ at a given time point (ti) divided by the cell index CI_nml_time_ at the normalization time point (nml_time). The slope is used to describe the steepness of a curve within a given time window (in our case: 70 h proliferation).

#### Clonogenic Assay (Proliferation)

The *in vitro* cell survival assay based on the ability of a single cell to grow into a colony was performed as described [49]. To investigate the ability of cancer cells to form colonies from a single cell, 500 cells of the investigated cell lines were seeded into each chamber of a 6-well plate and incubated at the described culture conditions for 8 days. Afterwards, colonies were fixed with glutaraldehyde (6.0 % v/v) and stained with crystal violet (0.5 % w/v) for 25 min. Excess staining solution was removed, the wells washed until clear and the plates were scanned. The developed colonies were counted using the Olympus IX83 software.

### GFP fluorescence measurement

Cell lines carrying GFP plasmids (Mel Juso GFP & Mel Juso NRN1-GFP) were seeded into a black 96 well plate with clear bottoms (Corning, New York, USA). Culture supernatant was transferred to fresh wells after defined times and measured using the ClarioStar plate reader (BMG Labtech, Ortenberg). Settings were as follows: excitation at 470 (± 15) nm, emission measured at 515 (± 20) nm.

### Spheroid formation assay

Cells were seeded into 96 well plate after coating with 100 µl 1 % agarose at 4 000 cells/100 µl. After incubation times of 72 h the resulting spheroids were imaged and their diameter measured using the Olympus IX83 software.

#### Migration assay

To assess migration, Boyden chambers were used, separated by polycarbonate filters with a pore size of 8 µm. Filters were coated in gelatine (5 mg/l) for improved attachment. 4 x 10^4^ cells were seeded into the top chamber in serum free medium, with the lower chamber containing fibroblast-conditioned medium (FCM). Boyden chambers were incubated at standard culture conditions for 4 h, after which the migrated cells on the lower side of the filter were fixed and stained. Through microscopy, 10 fields of vision per filter were counted for cells. Experiments were performed in triplicates.

### Immunofluorescence staining

The staining procedure was previously described [40]. Used antibodies and concentrations are listed in Table 4.

### Statistical analysis

Statistical analysis was performed using GraphPad Prism software (GraphPad Software, Inc., San Diego, CA). This software was also used to create the graphs. The results are calculated as the mean ± SEM (range) or percent. Comparison between groups was determined using Student’s t-test. A *p*-value < 0.05 (*: *p*<0.05) was considered statistically significant (n.s.: not significant).

## Results

### NRN1 functions as oncogene

**Fig. 1:**
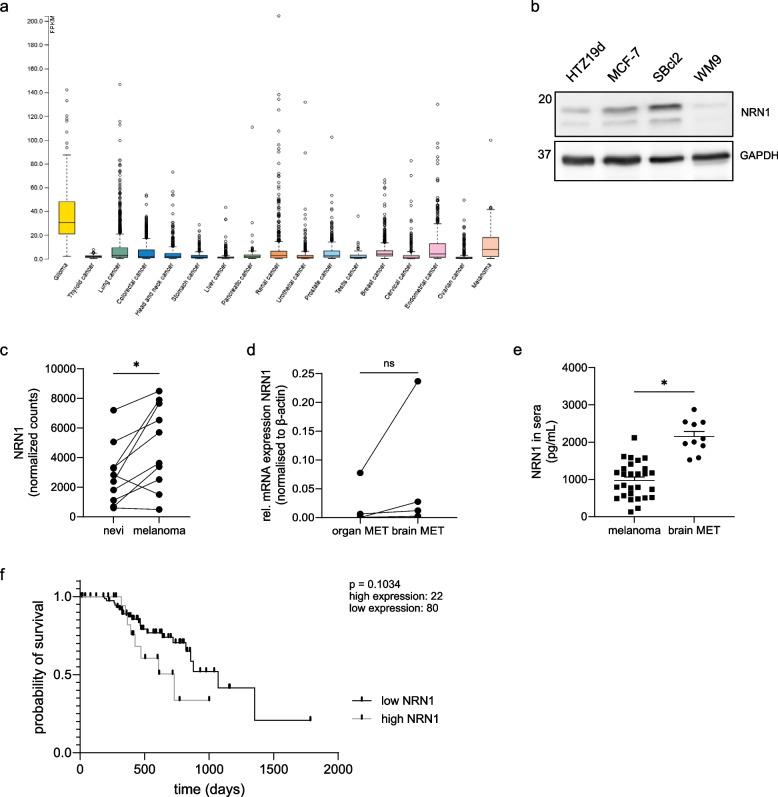
Neuritin-1 expression in different cancers, progression states and in vivo. **a** Comparison of NRN1 RNA expression based on TCGA (The Cancer Genome Atlas) data sets of different cancers. Fragments per kilo base of transcript per million mapped reads (FPKM) displayed for cancer entities. Minimum of 134 samples (testis cancer) and maximum of 1075 samples (breast cancer) per entity. **b** NRN1 protein expression in cancer cell lines. Western blot of basal NRN1 protein levels of cell lines HTZ19d, MCF-7, Sbcl2 and WM9. GAPDH was used as equal loading control. **c** NRN1 reads in RNA sequencing of nevi-melanoma pairs. Number of reads for 10 paired sample sets from nevi and melanoma site of patients. Data obtained from Kunz et al., 2018 (GSE112509). Graph displays individual values of paired samples. Two groups were statistically analysed using paired Students t-test. * = *p* < 0.05, ns = *p* > 0.05. **d** qRT-PCR expression analysis of NRN1 in paired organ and brain metastasis cell lines. mRNA expression was normalized to β-actin. Samples were provided by Dana Westphal, Dresden. Graph displays individual values of paired samples. Two groups were statistically analysed using paired Students t-test. * = *p* < 0.05, ns = *p* > 0.05. **e** NRN1 levels in sera of melanoma patients. Concentration measured through ELISA in sera of melanoma patients with or without brain metastases. Patient sera obtained from Annette Paschen and the Dermatological Department at FAU. Graph displays individual values along with mean ± SEM. Two groups were statistically analysed using unpaired Students t-test unless stated otherwise. * = *p* < 0.05, ns = *p* > 0.05. **f** Kaplan-Meier survival curve analysis was performed using the GEPIA2 database for a TCGA Skin Cutaneous Melanoma (SKCM) dataset. The survival curve is depicted for the total patient cohort separated into `low NRN1` (black) versus `high NRN1` (grey) group (*p* = 0.1034)

NRN1 is expressed in different cancer entities (https://www.proteinatlas.org/ENSG00000124785-NRN1/pathology; December 2023), to the highest degree in glioma, breast cancer and melanoma (Fig. 1a). As investigated in our previous publication NRN1 expression is higher in melanoma than in normal human epidermal melanocytes [10]. Within melanoma itself, NRN1 expression seems to be varied between different cell lines, shown exemplarily in Fig. 1b. Previous data also showed that there was no clear correlation between NRN1 protein levels and tumor stage in melanoma. Kunz et al. performed a comprehensive RNA-Seq analysis of laser-microdissected melanocytic nevi and primary melanoma samples derived from paired tissue samples of the same patient (*n* = 10) [50]. What could be shown in vivo, is an upregulation of NRN1 mRNA levels from nevi to primary melanoma (Fig. 1c). Therefore, NRN1 seems to play a role in early melanoma development.

Interestingly, there also seems to be a connection between NRN1 and melanoma brain metastasis. We analyzed the mRNA expression level of NRN1 by qRT-PCR in paired samples of primary cell lines generated out of brain metastasis and organ metastasis from tissues of the same patient [51]. The expression of NRN1 in melanoma brain metastasis is slightly elevated compared to organ metastasis (Fig. 1d). Furthermore, we analyzed serum from melanoma patients (*n* = 38) by ELISA measurement. NRN1 can be found in significantly increased levels in the serum of melanoma patients with brain metastases (Fig. 1e). In a cohort of melanoma TCGA (The Cancer Genome Atlas) data, a high expression of NRN1 leads to a decrease in probability of survival (Fig. 1f). In summary, these data point towards NRN1 playing an important oncogenic role in cancer, and melanoma especially.

**Fig. 2:**
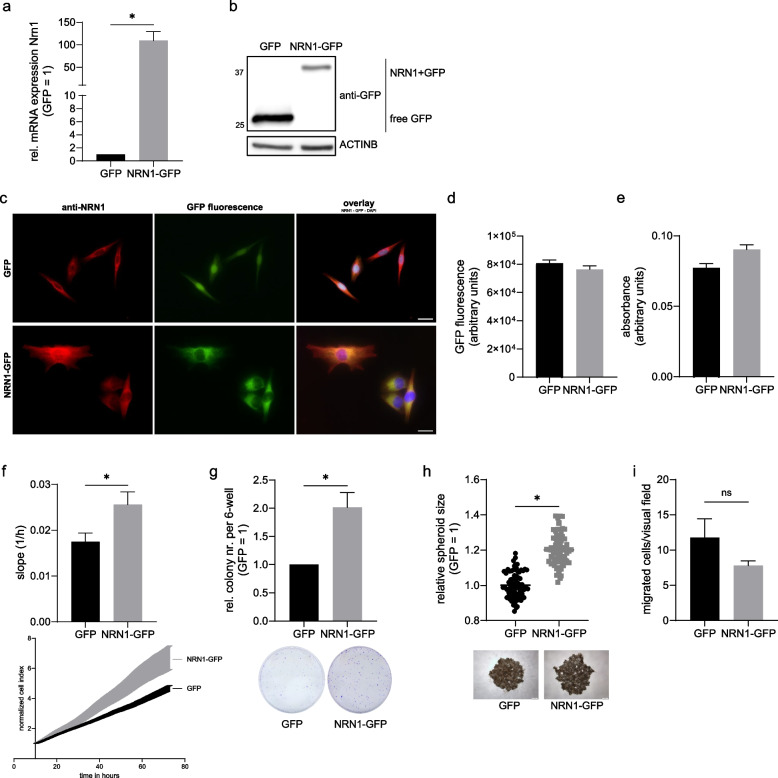
Characterization of overexpression cell line – NRN1 expression and functional analyses.** a** Analysis of NRN1 mRNA levels in GFP and NRN1-GFP cell lines through qRT-PCR. Expression levels normalized to housekeeper β-actin. GFP set to 1. **b** Protein expression validation of NRN1-GFP through Western blot. Example blot of GFP and NRN1-GFP cell line protein extracts, probed with GFP primary antibody. Equal loading was controlled with β-actin primary antibody. **c** Immunofluorescence of fixed cells against NRN1. Intrinsic GFP fluorescence (green) with NRN1 primary antibody staining (red) and overlay including nuclear DAPI staining (blue). The bar in the overlay represents 20 µm. **d** GFP fluorescence measurement in cell culture supernatant. GFP fluorescence was measured at 515 nm. **e** ELISA for NRN1 with cell culture supernatants. Absorbance of staining solution was measured at 450 nm. **f** Proliferation analysis of model cell lines using RTCA. Proliferation was compared by slope. Growth curves of normalized cell index for GFP and NRN1-GFP. **g** Clonogenic assay of GFP and NRN1-GFP. Analysis of clonogenic ability of model system cell lines. GFP set to 1. Representative images of colonies 8 days after seeding, fixed and stained using crystal violet. **h** Spheroid formation on agar. Comparison of spheroid diameter of GFP and NRN1-GFP cells after 72 h. GFP set to 1. Representative images of spheroids after 72 h. **i** Migration analysis of cell lines using Boyden chambers. Comparison of number of migrated cell per visual field for GFP and NRN1-GFP. All graphs are displayed as mean ± SEM. Two groups were statistically analysed using unpaired Students t-test unless stated otherwise. * = *p* < 0.05, ns = *p* > 0.05

To establish a model system of the elevated NRN1 expression in melanoma, we generated a stable overexpression cell line using the melanoma cell line Mel JuSo (with a comparatively low NRN1 expression). A CMV::NRN1-GFP (NRN1-GFP) construct or a CMV::GFP (GFP) control were transduced using a lentiviral system. The positive population was bulk sorted by its GFP signal by FACS (FACS core facility, FAU Erlangen). The overexpression and long-term stability were analysed on both the mRNA level for NRN1 (Fig. 2a) and indirectly on the protein level using a GFP antibody to detect both the free GFP in the control cell line as well as the (shifted by ~13 kDa) NRN1+GFP in the NRN1 overexpression cell line (Fig. 2b). To confirm the NRN1 protein overexpression more directly we also performed Western blotting using an antibody against NRN1 (Supplementary Figure 1a). Immunofluorescence analysis of the GFP fluorescence signal visualizes the NRN1 overexpression in the cytoplasm of the NRN1-GFP cell clone. By using a specific anti-NRN1 antibody we confirm an endogenous and cytoplasmic NRN1 expression in the GFP control and in the NRN1 overexpressing cell clone (red). The cytoplasmic NRN1 overexpression is underlined by the distinct overlap of the anti-NRN1 immunofluorescence with the GFP signal (orange-yellow) (Fig. 2c). The (NRN1-)GFP signal in the NRN1-GFP cell line was also localized differently than the free GFP in the control cell line. The NRN1-GFP protein did not enter the nucleus, but was rather found in the cytoplasm while still seeming to concentrate around the nucleus. Free GFP seemed to be located both in the nucleus and ubiquitously in the cytoplasm (Fig. 2c). Since NRN1 has been shown to be secreted as well, we investigated our model system for secretion of NRN1-GFP into the extracellular space. Neither the measurement of GFP fluorescence in cultured cell supernatants (Fig. 2d) nor the ELISA for NRN1 (Fig. 2e) showed any significant difference in extracellular NRN1 level, indicating that NRN1 was not secreted to a higher degree by the overexpression cell line. We therefore concluded that all functional and signaling effects were due to intracellular NRN1 alone. For functional validation of our model system, we performed experiments that had been used to investigate the cells’ functionality with short interfering RNA against NRN1 (siNRN1) in our previous publication [10]. An increase in proliferation was caused by NRN1-GFP overexpression (Fig. 2f), measured using the xCELLigence real time cell analysis (RTCA) system. In addition, we performed a clonogenic assay, in which a low number of cells is seeded to test each cell’s individual ability to divide “eternally” and thereby form a colony. In line with the increased proliferation rate, the NRN1-GFP cell clone showed an increased clonogenicity compared to the GFP control (Fig. 2g). To further investigate the direction of increased growth and replication potential, we performed a tumor spheroid formation assay in 3D cell culture. In line with the increased proliferation and clonogenicity, the NRN1-GFP cell clone spheroids were significantly larger than the GFP spheroids after 72 h (Fig. 2h). Analyzing the potential for migration in Boyden chamber assays, no significant difference was detectable between the control and NRN1-GFP cell clone (Fig. 2i). In summary, our established model system was able to show increased NRN1 expression leading to increased proliferation, clonogenicity and spheroid formation, all due to the signaling effects of intracellular NRN1 alone.

### NRN1 and its association with Notch signaling

**Fig. 3:**
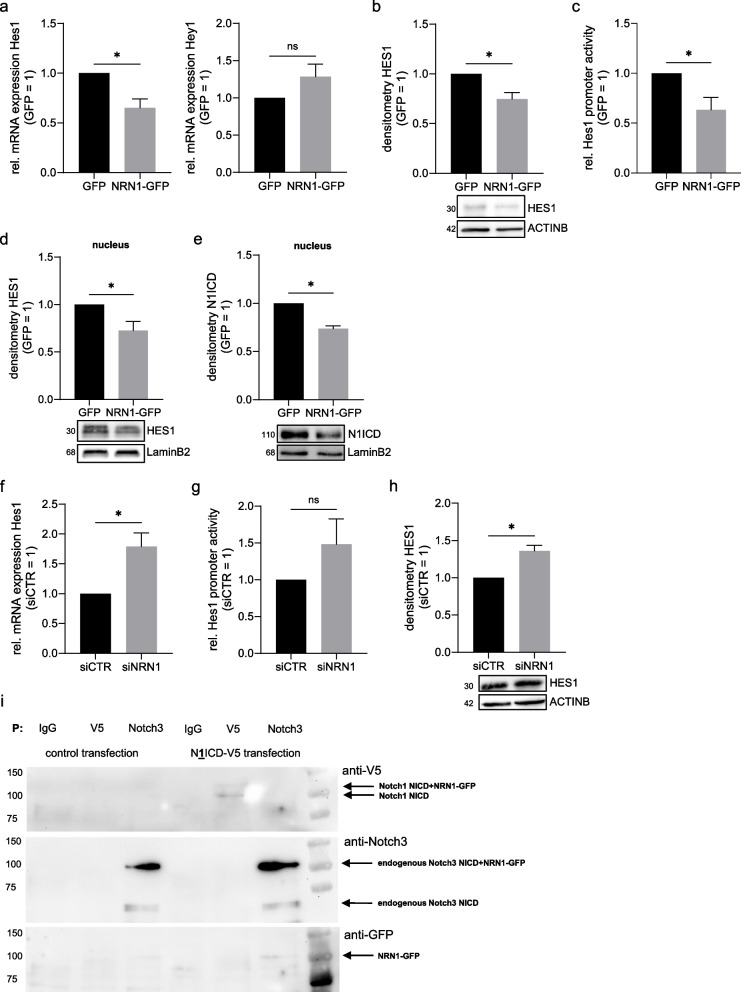
Notch signaling and downstream targets in overexpression cell line and under knock-down. **a** qRT-PCR of mRNA expression levels of Hes1 and Hey1. Comparison of Notch downstream target expression between GFP and NRN1-GFP. Expression levels normalized to β-actin. GFP set to 1. **b** Protein expression of HES1. Analysis of protein levels of HES1 in GFP and NRN1-GFP cell lines through Western blot. Equal loading was controlled with GAPDH primary antibody. GFP set to 1. **c** Luciferase assay for analysis of Hes1 promotor activity. Comparison of Hes1 promotor activity between GFP and NRN1-GFP. Measurements normalized to transfection control pRL-TK. GFP set to 1. **d** Protein expression of N1ICD in nuclear extracts. Western blot of nuclear fractions comparing GFP and NRN1-GFP. Equal loading was controlled with LaminB2 primary antibody. GFP set to 1. **e** Protein expression of HES1 in nuclear extracts. Western blot of nuclear fractions comparing GFP and NRN1-GFP. Equal loading was controlled with LaminB2 primary antibody. GFP set to 1. **f** Analysis of mRNA levels of Hes1 under siNRN1 knock-down. qRT-PCR comparing siCTR and siNRN1. Expression levels normalized to β-actin. siCTR set to 1. **g** Luciferase assay for analysis of Hes1 promotor activity under siNRN1 knock-down. Comparison of Hes1 promotor activity between siCTR and siNRN1. Measurements normalized to transfection control pRL-TK. siCTR set to 1. **h** Protein expression of HES1 under siNRN1 knock-down. Comparing protein levels of HES1 in siCTR and siNRN1 lysates through Western blot. Equal loading was controlled with β-actin primary antibody. siCTR set to 1.** i** Immunoprecipitation of NRN1-GFP lysates after transfection (control, N1ICD-V5). Lysates were pulled with IgG, V5 and Notch3 antibody. Western blot was probed with V5 primary antibody, Notch3 primary antibody and GFP primary antibody. Detected interaction complexes are denoted by black arrow. All graphs are displayed as mean ± SEM. Two groups were statistically analysed using unpaired Students t-test unless stated otherwise. * = *p* < 0.05, ns = *p* > 0.05

Trying to elucidate the molecular mechanisms of NRN1 signaling in melanoma cells, recent literature [14, 52, 53] has established a connection with Notch signaling. Using multiple different avenues of investigation, we evaluated the activity of Notch signaling in the NRN1-GFP overexpression cell line. mRNA levels of Notch downstream target *Hairy and Enhancer of Split 1* (HES1) showed a significant decrease in the NRN1-GFP cell clone compared to the control GFP cell clone using qRT-PCR (Fig. 3a). We also investigated the second classical downstream target of Notch, *Hes related family bHLH transcription factor with YRPW motif 1* (HEY1). Here, we could see no significant effect of the NRN1-GFP overexpression on HEY1 mRNA levels (Fig. 3a). We further confirmed the decreased levels of HES1 on both the protein level using Western blot (Fig. 3b) and on the direct transcriptional level (Fig. 3c) using a luciferase-based reporter system. Interestingly, the downregulation of HES1 protein could be shown clearly in nuclear fractions of cellular extract (Fig. 3d), which was also true for Notch1 intracellular domain (N1ICD), the classical Notch signaling effector (Fig. 3e). These experiments were then repeated using siNRN1 knockdown of the NRN1-GFP overexpression, to further confirm the effect was due to NRN1 directly. In the siNRN1 knockdown, Hes1 transcription, protein levels and promoter activity were all increased (Fig. 3f-h), confirming the inhibitory effect of NRN1 on Notch signaling in melanoma.

Focusing on a potential direct interaction between NRN1 and the Notch signaling pathway in the cytoplasm, we investigated whether NRN1 was able to bind to Notch intracellular domains. For immunoprecipitation experiments, pcDNA (control transfection) and N1ICD-V5 plasmids were transfected into NRN1-GFP cells. Protein lysates were then incubated with following antibodies bound to G-Sepharose beads: rabbit IgG as isotype control, V5 for precipitation of N1ICD-V5 bound proteins and Notch3 for precipitation of endogenous N3ICD along with any bound proteins. Western blots for separation of immunoprecipitated fractions were probed with GFP antibody for detection of NRN1-GFP. Subsequent probing with V5 and Notch3 antibody was used as both internal control for pulldown and to detect a potential shift of signal induced by binding of NRN1-GFP (Fig. 3i). In N1ICD-V5 transfected cells pulled with V5 we saw a signal at around 110 kDa using the V5 antibody on the Western blot, while we also saw that signal upon probing with NOTCH3 antibody. Through this we were able to show a direct interaction of NRN1 and NICD.

### NRN1 and its role in STAT3 signaling

**Fig. 4:**
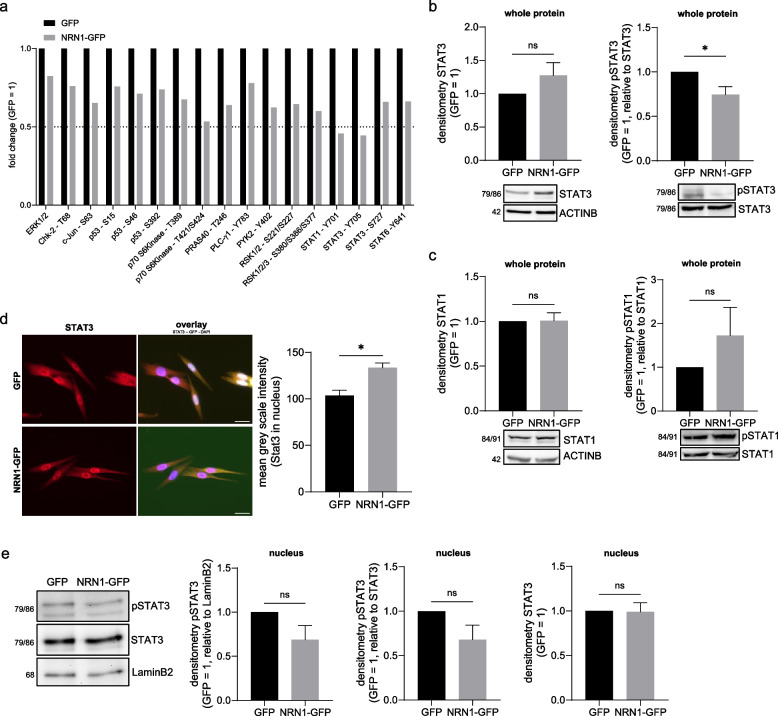
Influence of NRN1 overexpression on STATs and their phosphorylation status. **a** Downregulated targets of phosphospot array comparing GFP and NRN1-GFP lysates. Phosphorylation status of kinases and proteins with downregulation relative to GFP. Visualization line marks 0.5x fold change. GFP set to 1. **b** Protein expression levels of STAT3 and pSTAT3. Validation of phosphospot results using Western blot of whole cell lysates of GFP and NRN1-GFP. Equal loading for STAT3 was controlled by β-actin primary antibody. pSTAT3 expression was normalized to STAT3 expression. GFP set to 1. **c** Protein expression levels of STAT1 and pSTAT1 through Western blot. Comparison of STAT1 and pSTAT1 expression in whole cell lysates between GFP and NRN1-GFP. Equal loading for STAT1 was controlled by β-actin primary antibody. pSTAT1 expression was normalized to STAT1 expression. GFP set to 1. **d** Immunofluorescence of fixed cells against STAT3. GFP and NRN1-GFP with STAT3 primary antibody (red) and as overlay with intrinsic GFP (green) and nuclear stain DAPI (blue). Measurement of mean grey scale intensity of STAT3 fluorescence signal in nuclei (ROI defined by DAPI signal) comparing GFP with NRN1-GFP. **e** Protein expression analysis of pSTAT3 and STAT3 in nuclear extracts of GFP and NRN1-GFP. Western blot with pSTAT3 and STAT3 primary antibodies. LaminB2 primary antibody was used to control equal loading. Analysis of expression levels of pSTAT3 in nuclei, normalized to LaminB2 or STAT3 expression. Analysis of expression levels of STAT3 in nuclei, normalized to LaminB2. GFP set to 1. All graphs are displayed as mean ± SEM. Two groups were statistically analysed using unpaired Students t-test unless stated otherwise. * = *p* < 0.05, ns = *p* > 0.05

To further unravel NRN1-influenced signaling in melanoma and other malignancies, we used a pre-spotted array of important phosphorylated proteins involved in several different major cellular signaling pathways. Using cell lysate as specified in the manufacturer’s protocol, the GFP and NRN1-GFP cell clones were compared for the phosphorylation – and implied activation – of 37 different kinases and two additional proteins. AKT1/2/3, CREB, GSK or p38 are examples of proteins unchanged in their activity (data not shown). Interestingly, we found several phosphorylation sites of kinases to be downregulated in the NRN1-GFP line compared to the GFP control line (Fig. 4a). What piqued our interest was the fact that the phosphorylation on both sites of STAT3 – Y705 (50 % reduction of phosphorylation) and S727 – was downregulated, as well as Y701 on STAT1. We tried to confirm this first glimpse by independent Western blot analyses of both whole STAT3 and specific pY705-STAT3 in total protein lysate. Surprisingly, here we saw an overall upregulation of the total protein amount of STAT3 (Fig. 4b), whereas pY705-STAT3 was downregulated (Fig. 4b). In summary, the reduced phosphorylation of STAT3-Y705 after NRN1 overexpression, seen in the *phospho-kinase* *array* was confirmed and particularly reaffirmed, as the total protein content of STAT3 even increases.

We were however unable to confirm the downregulation of pSTAT1 of the *phospho-kinase array* through Western blot, instead seeing a potential upregulation in relation to whole STAT1 (Fig. 4c). Using immunofluorescence of GFP and NRN1-GFP cell clones for STAT3 we confirmed the upregulation of total STAT3 in the cytoplasm (Supplementary Figure 2a) and the nucleus (Fig. 4d). We sought to confirm these results in nuclear fractions of protein extracts as well using both the pSTAT3 and total STAT3 antibodies. Seeing as there was less phosphorylated STAT3 (both relative to LaminB2 and total STAT3; Fig. 4e) and an unchanged amount of total STAT3 (Fig. 4f), we investigated the possibility of unphosphorylated STAT3 (uSTAT3) being responsible for the observed changes and signaling in the NRN1-GFP overexpression cell line. For STAT1, only a small downregulation of pSTAT1 with unchanged levels of whole STAT1 could be shown (Supplementary Figure 2b). We therefore concluded that the effects were due to the STAT3 axis.

**Fig. 5:**
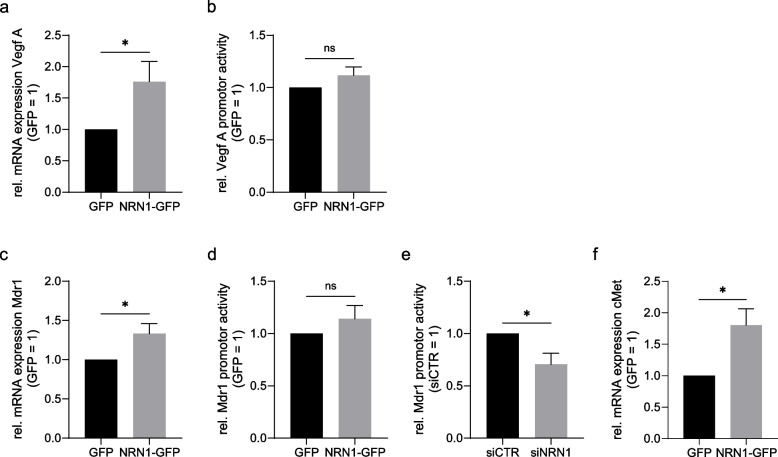
Expression of STAT3 targets under NRN1 overexpression. **a** qRT-PCR analysis of mRNA expression of Vegf A. Comparison of GFP and NRN1-GFP. Expression levels normalized to β-actin. GFP set to 1. **b** Vegf A promotor activity analysis. Luciferase-based assay comparing Vegf A promotor activity of GFP and NRN1-GFP cells. Measurements normalized to transfection control pRL-TK. GFP set to 1. **c** mRNA analysis of Mdr1 in GFP and NRN1-GFP cells through qRT-PCR. Expression levels normalized to β-actin. GFP set to 1. **d** Mdr1 promotor activity analysis using Luciferase-based assay comparing GFP and NRN1-GFP cells. Measurements normalized to transfection control pRL-TK. GFP set to 1. **e** Mdr1 promotor activity analysis using Luciferase-based assay comparing siCTR and siNRN1 knockdown cells. Measurements normalized to transfection control pRL-TK. siCTR set to 1. **f** cMet mRNA expression analysis using qRT-PCR of GFP and NRN1-GFP. GFP set to 1. All graphs are displayed as mean ± SEM. Two groups were statistically analysed using unpaired Students t-test unless stated otherwise. * = *p* < 0.05, ns = *p* > 0.05

Since the field of uSTAT3 signaling is still comparatively new and not well established in melanoma specifically, we analysed several different potential targets, which had already been described to be specific for uSTAT3 in other malignancies [35, 39]. We saw an upregulation of vascular endothelial growth factor A (VEGF A) on both mRNA and promotor level (Fig. 5a, b). Similarly, MDR1 was also upregulated based in mRNA (Fig. 5c) and promotor analysis (Fig. 5d), where the latter was reversible by siNRN1 knockdown in NRN1-GFP overexpression cell line (Fig. 5e). On mRNA level alone, we could see an upregulation of cMet (Fig. 5f) in the NRN1-GFP overexpression cell line. Through these downstream targets, we were able to confirm an upregulation of the non-canonical uSTAT3 signaling linked to NRN1-GFP overexpression. Interestingly, JAB1 could be involved in this target gene regulation by NRN1, since JAB1 could be a binding partner for uSTAT3 in the nucleus to regulate the transcription of target genes. Our data showed that NRN1 also induces JAB1 protein levels (Supplementary Figure 2c).

## Discussion

Since malignant melanoma represents a tumor derived from a unique lineage, sharing the ancestry of the neural crest with other cell types such as neurons, the investigation of cellular programming causing the switch to malignancy has often been driven in this direction. The protein NRN1 (also CPG15) was first identified in a screen investigating genes related to neuronal plasticity [4]. The first data regarding NRN1 showed an effect on neurite regeneration and dendrite outgrowth [54, 55]. This protein has since been implicated in several different cancers, where it was found to be overexpressed [56]. Since we could already show this overexpression of NRN1 in melanoma [10], we used an intracellular overexpression system to elucidate some of the signaling pathways and cellular functions NRN1 is involved in. Since our *in vitro* model did not lead to increased secretion of NRN1, all effects investigated were due to intracellular NRN1. More availability of NRN1 intracellularly did not seem to lead to an upregulation of the secretion apparatus of the cells.

Notch signaling has already been implicated in several different malignancies, with varying outcomes, however. This signaling pathway has been found to act in both a tumor suppressive [57] and an oncogenic way [58] in different cancers. The regulation and downstream effects of Notch are highly tissue specific and depend on cellular context in general. The Notch signaling pathway exerts its effects through two main transcription factors, HES1 and HEY1, which act as transcriptional repressors for a multitude of genes. We focused our investigations on HES1 and HEY1 and not on the other existing variants, since they are the most transcribed variants in human cells and cancers [59] and are generally the most expressed in melanoma cell lines specifically [18]. HES1 and HEY1 take on different roles through binding of different corepressors [60] as well as differences in the preference for DNA binding sites [60, 61]. Our data showing a downregulation of Notch signaling along the N1ICD-HES1 axis due to high NRN1 expression astonishingly suggest a tumour-suppressive role of the NRN1-Notch-Hes1 interaction in the skin and melanoma development especially [57].

The STAT signaling axis has been well studied in cancers, especially its activation through phosphorylation and subsequent dimerization. The constitutive activation of this pathway has been shown to contribute to essential characteristics of malignant cells, like proliferation, survival and epithelial-mesenchymal transition [62]. Apart from the classical activation through tyrosine phosphorylation, other modes of cellular regulation have emerged. First among them, phosphorylation of serine 727 on STAT3 leads to a rerouting of cellular localization from the nucleus to the mitochondria [63]. Another alternative signaling pathway has been defined as the regulation of gene expression through unphosphorylated STAT3 [35, 38]. This has been shown to occur as a second wave of gene expression after canonical STAT3 activation through IL6 stimulation [35], targeting a unique subset of genes such as mRas and cMet. In another study, uSTAT3 was shown to interact with JAB1 for pSTAT3 independent regulation of genes like MDR1, Nanog and VEGF [39]. According to our findings of reduced pSTAT3 levels in our NRN1 overexpression model, we investigated the role of NRN1-modulated uSTAT3 signaling and found an increase of uSTAT3 specific target genes. We did also investigate the role of STAT1, since its phosphorylation was shown to be downregulated in the *phospho-kinase array* as well. However, we couldn’t find any significant difference in pSTAT1 or STAT1 levels, both in whole cell extracts or nuclear extracts, leaving us to conclude that the effects observed were mainly due to uSTAT3.

Previous studies have already shown a direct interaction between the Notch and STAT signaling pathways [23, 64, 65]. In COS-1 cells, HES1 was found to increase the phosphorylation of STAT3 through binding and subsequent recruitment of the responsible kinase JAK1 [23]. Considering the fact that in our model system of high NRN1 expression in melanoma we saw a direct binding of NRN1 to N1ICD and N3ICD, we hypothesize a retention of the Notch intracellular domains in the cytoplasm through NRN1. This then causes the decrease in nuclear NICD levels, consequently leading to the decrease in HES1 transcription. Similarly to the mechanism described in the previously mentioned publication, the NRN1-induced lower HES1 levels would therefore lead to a decrease in STAT3 phosphorylation through lack of binding and more uSTAT3 signaling as observed in our model system.

### Supplementary Information

**Additional file 1:** **Supplementary Figure 1.** a: Analysis of NRN1 protein levels in GFP and NRN1-GFP cell lines through Western blot. Immunoblot probed with primary NRN1 antibody Expression levels normalized to primary β-actin antibody. GFP set to 1. *n* = 2. Graph is displayed as mean ± SEM. Two groups were statistically analysed using unpaired Students t-test unless stated otherwise. * = *p* < 0.05, ns = *p* > 0.05.

**Additional file 2:** **Supplementary Figure 2.** a Immunofluorescence of fixed cells against STAT3. Measurement of mean grey scale intensity of STAT3 fluorescence signal in cytoplasm comparing GFP with NRN1-GFP. b: Protein expression analysis of pSTAT1 and STAT1 in nuclear extracts of GFP and NRN1-GFP. Western blot with pSTAT1 and STAT1 primary antibodies. LaminB2 primary antibody was used to control equal loading. Analysis of expression levels of pSTAT1 in nuclei, normalized to LaminB2. Analysis of expression levels of STAT1 in nuclei, normalized to LaminB2. GFP set to 1. c: Protein expression of JAB1 through Western blot. Example blot of GFP and NRN1-GFP cell line protein extracts, probed with JAB1 primary antibody. Equal loading was controlled with β-actin primary antibody. All graphs are displayed as mean ± SEM. Two groups were statistically analysed using unpaired Students t-test unless stated otherwise. * = *p* < 0.05, ns = *p* > 0.05.

## Data Availability

No datasets were generated or analysed during the current study.

## Abbreviations

NRN1: Neuritin-1; cpg15, candidate-plasticity gene 15
STAT: Signal transducer and activator of transcription
NICD: Notch intracellular domain
GFP: Green fluorescent protein
FACS: Fluorescence-activated cell sorting
HES1: Hairy and Enhancer of Split 1
HEY1: Hes Related Family BHLH Transcription Factor With YRPW Motif 1
VEGF A: Vascular endothelial growth factor A
TrkA: Tyrosine kinase A
JAB1: Jun activation domain-binding protein 1
qRT-PCR: Quantitative real time polymerase chain reaction
RIPA: Radioimmunoprecipitation Assay
MP: Milk powder
BSA: Bovine serum albumin
HRP: Horseradish peroxidase
IP: Immunoprecipitation
ELISA: Enzyme linked immunosorbent assay
TCGA: The Cancer Genome Atlas
RTCA: Real time cell analysis

## References

[1] Moschos SJ (2022). Melanoma brain metastases: an update on the use of immune checkpoint inhibitors and molecularly targeted agents. Am J Clin Dermatol.

[2] Patton EE, Mueller KL, Adams DJ, Anandasabapathy N, Aplin AE, Bertolotto C (2021). Melanoma models for the next generation of therapies. Cancer Cell.

[3] Artinger KB, Monsoro-Burq AH (2021). Neural crest multipotency and specification: power and limits of single cell transcriptomic approaches. Fac Rev.

[4] Nedivi E, Hevroni D, Naot D, Israeli D, Citri Y (1993). Numerous candidate plasticity-related genes revealed by differential cDNA cloning. Nature.

[5] Naeve GS, Ramakrishnan M, Kramer R, Hevroni D, Citri Y, Theill LE (1997). Neuritin: a gene induced by neural activity and neurotrophins that promotes neuritogenesis. Proc Natl Acad Sci U S A.

[6] Le Jan S, Le Meur N, Cazes A, Philippe J, Le Cunff M, Leger J (2006). Characterization of the expression of the hypoxia-induced genes neuritin, TXNIP and IGFBP3 in cancer. FEBS Lett.

[7] Yuan M, Li Y, Zhong C, Li Y, Niu J, Gong J (2015). Overexpression of neuritin in gastric cancer. Oncol Lett.

[8] Han D, Qin B, Liu G, Liu T, Ji G, Wu Y (2011). Characterization of neuritin as a novel angiogenic factor. Biochem Biophys Res Commun.

[9] Zhang L, Zhao Y, Wang CG, Fei Z, Wang Y, Li L (2011). Neuritin expression and its relation with proliferation, apoptosis, and angiogenesis in human astrocytoma. Med Oncol.

[10] Bosserhoff AK, Schneider N, Ellmann L, Heinzerling L, Kuphal S (2017). The neurotrophin Neuritin1 (cpg15) is involved in melanoma migration, attachment independent growth, and vascular mimicry. Oncotarget.

[11] Yao JJ, Zhao QR, Lu JM, Mei YA (2018). Functions and the related signaling pathways of the neurotrophic factor neuritin. Acta Pharmacol Sin.

[12] Yao JJ, Gao XF, Chow CW, Zhan XQ, Hu CL, Mei YA (2012). Neuritin activates insulin receptor pathway to up-regulate Kv4.2-mediated transient outward K+ current in rat cerebellar granule neurons. J Biol Chem.

[13] Karamoysoyli E, Burnand RC, Tomlinson DR, Gardiner NJ (2008). Neuritin mediates nerve growth factor-induced axonal regeneration and is deficient in experimental diabetic neuropathy. Diabetes.

[14] Zhang P, Luo X, Guo Z, Xiong A, Dong H, Zhang Q (2017). Neuritin inhibits notch signaling through interacted with neuralized to promote the neurite growth. Front Mol Neurosci.

[15] Jain CK, Bhargava S, Jain I, Varshney S (2022). Targeting notch pathway in cancer diagnostics and therapeutics: an emerging approach. Recent Pat Anticancer Drug Discov.

[16] Ferreira A, Aster JC (2022). Notch signaling in cancer: complexity and challenges on the path to clinical translation. Semin Cancer Biol.

[17] Aitini E, Cavazzini G, Cantore M, Rabbi C, Malaspina R, Truzzi R (1995). Carboplatin and etoposide in an out-patient schedule for the palliation of advanced non-small-cell lung cancer. Tumori.

[18] Balint K, Xiao M, Pinnix CC, Soma A, Veres I, Juhasz I (2005). Activation of Notch1 signaling is required for beta-catenin-mediated human primary melanoma progression. J Clin Invest.

[19] Bonyadi Rad E, Hammerlindl H, Wels C, Popper U, Ravindran Menon D, Breiteneder H (2016). Notch4 signaling induces a mesenchymal-epithelial-like transition in melanoma cells to suppress malignant behaviors. Cancer Res.

[20] Liu ZJ, Xiao M, Balint K, Smalley KS, Brafford P, Qiu R (2006). Notch1 signaling promotes primary melanoma progression by activating mitogen-activated protein kinase/phosphatidylinositol 3-kinase-Akt pathways and up-regulating N-cadherin expression. Cancer Res.

[21] Mikheil DM, Prabhakar K, Arshad A, Rodriguez CI, Newton MA, Setaluri V (2019). Notch signaling activation induces cell death in MAPKi-resistant melanoma cells. Pigment Cell Melanoma Res.

[22] Hu YY, Zheng MH, Zhang R, Liang YM, Han H (2012). Notch signaling pathway and cancer metastasis. Adv Exp Med Biol.

[23] Kamakura S, Oishi K, Yoshimatsu T, Nakafuku M, Masuyama N, Gotoh Y (2004). Hes binding to STAT3 mediates crosstalk between Notch and JAK-STAT signalling. Nat Cell Biol.

[24] Tulip IJ, Kim SO, Kim EJ, Kim J, Lee JY, Kim H (2021). Combined inhibition of STAT and Notch signalling effectively suppresses tumourigenesis by inducing apoptosis and inhibiting proliferation, migration and invasion in glioblastoma cells. Anim Cells Syst (Seoul).

[25] Wenta N, Strauss H, Meyer S, Vinkemeier U (2008). Tyrosine phosphorylation regulates the partitioning of STAT1 between different dimer conformations. Proc Natl Acad Sci U S A.

[26] Bohm M, Schulte U, Funk JO, Raghunath M, Behrmann I, Kortylewski M (2001). Interleukin-6-resistant melanoma cells exhibit reduced activation of STAT3 and lack of inhibition of cyclin E-associated kinase activity. J Invest Dermatol.

[27] Kreis S, Munz GA, Haan S, Heinrich PC, Behrmann I (2007). Cell density dependent increase of constitutive signal transducers and activators of transcription 3 activity in melanoma cells is mediated by Janus kinases. Mol Cancer Res.

[28] Fofaria NM, Srivastava SK (2014). Critical role of STAT3 in melanoma metastasis through anoikis resistance. Oncotarget.

[29] Grabner B, Moll HP, Casanova E (2016). Unexpected oncosuppressive role for STAT3 in KRAS-induced lung tumorigenesis. Mol Cell Oncol..

[30] Pencik J, Schlederer M, Gruber W, Unger C, Walker SM, Chalaris A (2015). STAT3 regulated ARF expression suppresses prostate cancer metastasis. Nat Commun.

[31] Musteanu M, Blaas L, Mair M, Schlederer M, Bilban M, Tauber S (2010). Stat3 is a negative regulator of intestinal tumor progression in Apc(Min) mice. Gastroenterology.

[32] Yang J, Stark GR (2008). Roles of unphosphorylated STATs in signaling. Cell Res.

[33] Liu L, McBride KM, Reich NC (2005). STAT3 nuclear import is independent of tyrosine phosphorylation and mediated by importin-alpha3. Proc Natl Acad Sci U S A.

[34] Darnell JE (1997). STATs and gene regulation. Science.

[35] Yang J, Liao X, Agarwal MK, Barnes L, Auron PE, Stark GR (2007). Unphosphorylated STAT3 accumulates in response to IL-6 and activates transcription by binding to NFkappaB. Genes Dev.

[36] Srivastava J, DiGiovanni J (2016). Non-canonical Stat3 signaling in cancer. Mol Carcinog.

[37] Nkansah E, Shah R, Collie GW, Parkinson GN, Palmer J, Rahman KM (2013). Observation of unphosphorylated STAT3 core protein binding to target dsDNA by PEMSA and X-ray crystallography. FEBS Lett.

[38] Yang J, Chatterjee-Kishore M, Staugaitis SM, Nguyen H, Schlessinger K, Levy DE (2005). Novel roles of unphosphorylated STAT3 in oncogenesis and transcriptional regulation. Cancer Res.

[39] Nishimoto A, Kugimiya N, Hosoyama T, Enoki T, Li TS, Hamano K (2013). JAB1 regulates unphosphorylated STAT3 DNA-binding activity through protein-protein interaction in human colon cancer cells. Biochem Biophys Res Commun.

[40] Seefried F, Haller L, Fukuda S, Thongmao A, Schneider N, Utikal J (2022). Nuclear AREG affects a low-proliferative phenotype and contributes to drug resistance of melanoma. Int J Cancer.

[41] Niessner H, Schmitz J, Tabatabai G, Schmid AM, Calaminus C, Sinnberg T (2016). PI3K pathway inhibition achieves potent antitumor activity in melanoma brain metastases in vitro and in vivo. Clin Cancer Res.

[42] Vetma V, Gutta C, Peters N, Praetorius C, Hutt M, Seifert O (2020). Convergence of pathway analysis and pattern recognition predicts sensitization to latest generation TRAIL therapeutics by IAP antagonism. Cell Death Differ.

[43] Nishimura M, Isaka F, Ishibashi M, Tomita K, Tsuda H, Nakanishi S (1998). Structure, chromosomal locus, and promoter of mouse Hes2 gene, a homologue of drosophila hairy and enhancer of split. Genomics.

[44] Hu Z, Jin S, Scotto KW (2000). Transcriptional activation of the MDR1 gene by UV irradiation. Role of NF-Y and Sp1. J Biol Chem.

[45] Wood LW, Cox NI, Phelps CA, Lai SC, Poddar A, Talbot C (2016). Thyroid Transcription Factor 1 Reprograms Angiogenic Activities of Secretome. Sci Rep.

[46] Martz CA, Ottina KA, Singleton KR, Jasper JS, Wardell SE, Peraza-Penton A (2014). Systematic identification of signaling pathways with potential to confer anticancer drug resistance. Sci Signal.

[47] Hu Z, Jin S, Scotto KW. Transcriptional activation of the MDR1 gene by UV irradiation. Role of NF-Y and Sp1. J Biol Chem. 2000;275(4):2979-85. 10.1074/jbc.275.4.2979 10644769

[48] Pieger K, Schmitt V, Gauer C, Giessl N, Prots I, Winner B, et al. Translocation of distinct alpha synuclein species from the nucleus to neuronal processes during neuronal differentiation. Biomolecules. 2022;12(8). 10.3390/biom12081108 PMC9406079 36009004

[49] Franken NA, Rodermond HM, Stap J, Haveman J, van Bree C (2006). Clonogenic assay of cells in vitro. Nat Protoc.

[50] Kunz M, Loffler-Wirth H, Dannemann M, Willscher E, Doose G, Kelso J (2018). RNA-seq analysis identifies different transcriptomic types and developmental trajectories of primary melanomas. Oncogene.

[51] Westphal D, Meinhardt M, Grutzmann K, Schone L, Steininger J, Neuhaus LT (2023). Identification of epigenetically regulated genes distinguishing intracranial from extracranial melanoma metastases. J Invest Dermatol.

[52] Zhang Q, Zhang J, Zhang J, Aerxiding P, Quhai A, Chen C (2019). The biological-behavioral effect of neuritin on non-small cell lung cancer vascular endothelial cells via VEGFR and Notch1. Onco Targets Ther.

[53] Yang L, Wang X, Sun J, Liu C, Li G, Zhu J (2021). Neuritin promotes angiogenesis through inhibition of DLL4/Notch signaling pathway. Acta Biochim Biophys Sin (Shanghai).

[54] Nedivi E, Fieldust S, Theill LE, Hevron D (1996). A set of genes expressed in response to light in the adult cerebral cortex and regulated during development. Proc Natl Acad Sci U S A.

[55] Nedivi E, Wu GY, Cline HT (1998). Promotion of dendritic growth by CPG15, an activity-induced signaling molecule. Science.

[56] Dong H, Luo X, Niu Y, Yu N, Gao R, Wang H (2018). Neuritin 1 expression in human normal tissues and its association with various human cancers. Int J Clin Exp Pathol.

[57] Nicolas M, Wolfer A, Raj K, Kummer JA, Mill P, van Noort M (2003). Notch1 functions as a tumor suppressor in mouse skin. Nat Genet.

[58] Ellisen LW, Bird J, West DC, Soreng AL, Reynolds TC, Smith SD (1991). TAN-1, the human homolog of the Drosophila notch gene, is broken by chromosomal translocations in T lymphoblastic neoplasms. Cell.

[59] Katoh M, Katoh M (2007). Integrative genomic analyses on HES/HEY family: Notch-independent HES1, HES3 transcription in undifferentiated ES cells, and Notch-dependent HES1, HES5, HEY1, HEY2, HEYL transcription in fetal tissues, adult tissues, or cancer. Int J Oncol.

[60] Iso T, Kedes L, Hamamori Y (2003). HES and HERP families: multiple effectors of the Notch signaling pathway. J Cell Physiol.

[61] Fischer A, Gessler M (2007). Delta-Notch–and then? Protein interactions and proposed modes of repression by Hes and Hey bHLH factors. Nucleic Acids Res.

[62] Avalle L, Pensa S, Regis G, Novelli F, Poli V (2012). STAT1 and STAT3 in tumorigenesis: a matter of balance. JAKSTAT.

[63] Macias E, Rao D, Carbajal S, Kiguchi K, DiGiovanni J (2014). Stat3 binds to mtDNA and regulates mitochondrial gene expression in keratinocytes. J Invest Dermatol.

[64] Jin S, Mutvei AP, Chivukula IV, Andersson ER, Ramskold D, Sandberg R (2013). Non-canonical Notch signaling activates IL-6/JAK/STAT signaling in breast tumor cells and is controlled by p53 and IKKalpha/IKKbeta. Oncogene.

[65] Francis SM, Larsen JE, Pavey SJ, Bowman RV, Hayward NK, Fong KM (2009). Expression profiling identifies genes involved in emphysema severity. Respir Res.

[66] Husain B, Kirchberger MC, Erdmann M, Schupferling S, Abolhassani AR, Frohlich W (2021). Inflammatory markers in autoimmunity induced by checkpoint inhibitors. J Cancer Res Clin Oncol.

