# Supplementary material for: NRN1 interacts with Notch to increase oncogenic STAT3 signaling in melanoma
Source: Cell Commun Signal. 2024 May 6;22:256. doi: 10.1186/s12964-024-01632-8 (PMC11071257; doi:10.1186/s12964-024-01632-8)
Supplement: Supplementary file 1 — Additional file 1: Supplementary Figure 1. a: Analysis of NRN1 protein levels in GFP and NRN1-GFP cell lines through Western blot. Immunoblot probed with primary NRN1 antibody Expression levels normalized to primary β-actin antibody. GFP set to 1. n = 2. Graph is displayed as mean ± SEM. Two groups were statistically analysed using unpaired Students t-test unless stated otherwise. * = p < 0.05, ns = p > 0.05. [file 12964_2024_1632_MOESM1_ESM.pdf]

Supplementary Figure 1

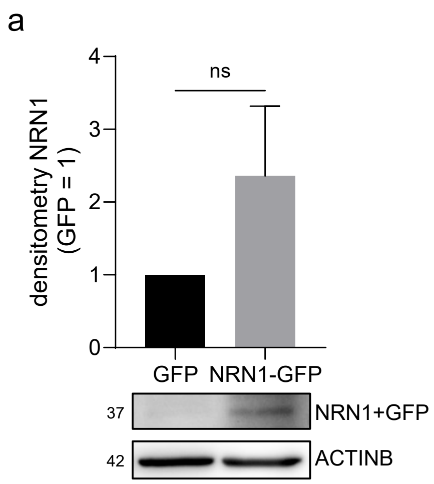

Supplementary Figure 1: a: Analysis of NRN1 protein levels in GFP and NRN1-GFP cell lines through Western Blot. Immunoblot probed with primary NRN1 antibody Expression levels normalized to primary β-actin antibody. GFP set to 1. n = 2. Graph is displayed as mean ± SEM. Two groups were statistically analysed using unpaired Students t-test unless stated otherwise. * = $p < 0.05$, ns = $p > 0.05$.
