# Supplementary material for: NRN1 interacts with Notch to increase oncogenic STAT3 signaling in melanoma
Source: Cell Commun Signal. 2024 May 6;22:256. doi: 10.1186/s12964-024-01632-8 (PMC11071257; doi:10.1186/s12964-024-01632-8)
Supplement: Supplementary file 2 — Additional file 2: Supplementary Figure 2. a Immunofluorescence of fixed cells against STAT3. Measurement of mean grey scale intensity of STAT3 fluorescence signal in cytoplasm comparing GFP with NRN1-GFP. b: Protein expression analysis of pSTAT1 and STAT1 in nuclear extracts of GFP and NRN1-GFP. Western blot with pSTAT1 and STAT1 primary antibodies. LaminB2 primary antibody was used to control equal loading. Analysis of expression levels of pSTAT1 in nuclei, normalized to LaminB2. Analysis of expression levels of STAT1 in nuclei, normalized to LaminB2. GFP set to 1. c: Protein expression of JAB1 through Western blot. Example blot of GFP and NRN1-GFP cell line protein extracts, probed with JAB1 primary antibody. Equal loading was controlled with β-actin primary antibody. All graphs are displayed as mean ± SEM. Two groups were statistically analysed using unpaired Students t-test unless stated otherwise. * = p < 0.05, ns = p > 0.05. [file 12964_2024_1632_MOESM2_ESM.pdf]

Supplementary Figure 2

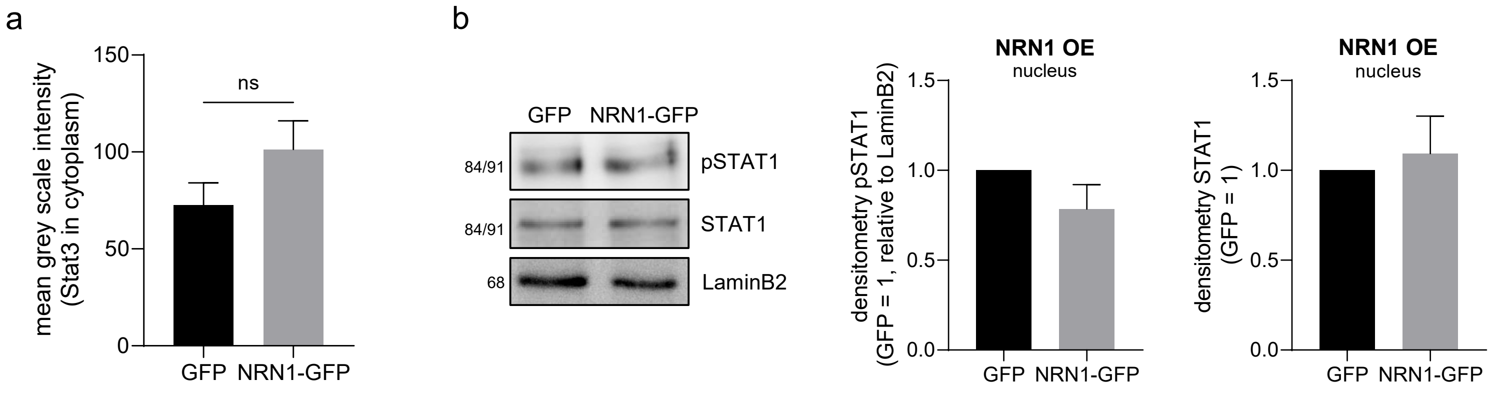

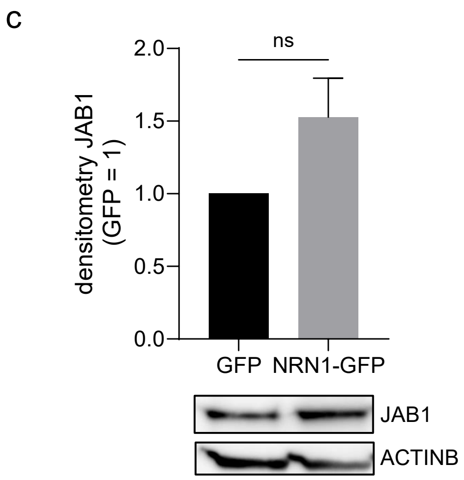

Supplementary Figure 2: a: Immunofluorescence of fixed cells against Stat3. Measurement of mean grey scale intensity of Stat3 fluorescence signal in cytoplasm comparing GFP with NRN1-GFP. **b:** Protein expression analysis of pStat1 and Stat1 in nuclear extracts of GFP and NRN1-GFP. Western Blot with pStat1 and Stat1 primary antibodies. LaminB2 primary antibody was used to control equal loading. Analysis of expression levels of pStat1 in nuclei, normalized to LaminB2. Analysis of expression levels of Stat1 in nuclei, normalized to LaminB2. GFP set to 1. **c:** Protein expression of JAB1 through Western Blot. Example blot of GFP and NRN1-GFP cell line protein extracts, probed with JAB1 primary antibody. Equal loading was controlled with β-actin primary antibody. All graphs are displayed as mean ± SEM. Two groups were statistically analysed using unpaired Students t-test unless stated otherwise. * = $p < 0.05$, ns = $p > 0.05$.
